# A social cost-benefit analysis of two One Health interventions to prevent toxoplasmosis

**DOI:** 10.1371/journal.pone.0216615

**Published:** 2019-05-10

**Authors:** Anita W. M. Suijkerbuijk, Eelco A. B. Over, Marieke Opsteegh, Huifang Deng, Paul F. van Gils, Axel A. Bonačić Marinović, Mattijs Lambooij, Johan J. Polder, Talitha L. Feenstra, Joke W. B. van der Giessen, G. Ardine de Wit, Marie-Josee J. Mangen

**Affiliations:** 1 National Institute for Public Health and the Environment, Bilthoven, the Netherlands; 2 Tilburg University, Tranzo, School of Social and Behavioral Sciences, Tilburg, the Netherlands; 3 University of Groningen, Department of epidemiology, Groningen, the Netherlands; 4 University Medical Center Utrecht, Julius Center for Health Sciences and Primary Care, Utrecht, the Netherlands; University of Illinois, UNITED STATES

## Abstract

In the Netherlands, toxoplasmosis ranks second in disease burden among foodborne pathogens with an estimated health loss of 1,900 Disability Adjusted Life Years and a cost-of-illness estimated at €45 million annually. Therefore, effective and preferably cost-effective preventive interventions are warranted. Freezing meat intended for raw or undercooked consumption and improving biosecurity in pig farms are promising interventions to prevent *Toxoplasma gondii* infections in humans. Putting these interventions into practice would expectedly reduce the number of infections; however, the net benefits for society are unknown. Stakeholders bearing the costs for these interventions will not necessary coincide with the ones having the benefits. We performed a Social Cost-Benefit Analysis to evaluate the net value of two potential interventions for the Dutch society. We assessed the costs and benefits of the two interventions and compared them with the current practice of education, especially during pregnancy. A ‘minimum scenario’ and a ‘maximum scenario’ was assumed, using input parameters with least benefits to society and input parameters with most benefits to society, respectively. For both interventions, we performed different scenario analyses. The freezing meat intervention was far more effective than the biosecurity intervention. Despite high freezing costs, freezing two meat products: steak tartare and mutton leg yielded net social benefits in both the minimum and maximum scenario, ranging from €10.6 million to €31 million for steak tartare and €0.6 million to €1.5 million for mutton leg. The biosecurity intervention would result in net costs in all scenarios ranging from €1 million to €2.5 million, due to high intervention costs and limited benefits. From a public health perspective (i.e. reducing the burden of toxoplasmosis) and the societal perspective (i.e. a net benefit for the Dutch society) freezing steak tartare and leg of mutton is to be considered.

## Introduction

The protozoan parasite *Toxoplasma gondii* (further referred to as *T*. *gondii*) is infecting a third of the human population globally and may cause toxoplasmosis [1]. Acquired toxoplasmosis in immunocompetent persons in general occurs without clinical symptoms, while in immunocompromised individuals uncontrolled multiplication of the parasite can have severe and potentially fatal implications, such as encephalitis [2]. During pregnancy, the parasite might be transmitted via the placenta and infect the fetus with varying severity from asymptomatic infection to life-threatening risk for the fetus and infant [2].

Cats are the definitive hosts of *T*. *gondii*: infected cats spread the parasite via oocysts excreted in their feces infecting warm blooded animals and humans either via direct contact or indirectly via the environment [3]. In livestock, toxoplasmosis mostly goes unnoticed as asymptomatic, but especially pregnant sheep and goats may have an abortion [4, 5]. However, tissue cysts can develop in all organs and tissues, including muscles of meat producing animals. Consumption of undercooked or raw meat products is therefore an important exposure pathway for humans. Humans can also become infected by ingestion of cat-shed oocysts via contaminated water or food, or via direct contact with cat feces [2, 6]. Additionally, congenital transmission can occur after primary infection during pregnancy [3, 6].

In the Netherlands, toxoplasmosis ranks second in disease burden among 14 foodborne diseases with in total 1,900 Disability Adjusted Life Years (DALY) in 2017, and additional cost-of-illnesses of €45 million [7, 8]. Given the burden of disease associated with *T*. *gondii*, effective and cost-effective preventive interventions are warranted. In the Netherlands, with an observed seroprevalence of 18.5% in women of reproductive age [9], toxoplasmosis prevention is targeted at education during pregnancy [10], similar to most other western European countries [11]. However, these interventions do not prevent acquisition of infections in the general population, whereby exposure via food (~56% of all symptomatic *T*. *gondii* infections in the Netherlands) is considered to be the most important route of infection [8]. Opsteegh et al. (2015) suggested—based on a quantitative risk assessment model—that freezing of raw consumed meat products would be effective to reduce the burden of disease as freezing meat at −20°C for 2 days eliminates infectious *T*. *gondii* tissue cysts [10, 12]. Other potential intervention measures are improving biosecurity to reduce exposure of the meat-producing animals to oocysts, and improved education to pregnant women and the general public [13]. Improved biosecurity on pig farms is considered an important factor in the decrease of seroprevalence observed in human populations [10]. The European Food Safety Authority (EFSA) working group has recommended a number of controlled housing conditions to prevent Toxoplasma infection in pigs [14] such as keeping cats away from stables and feed and implementing strict vermin control. Stringent biosecurity measures might result in a lower prevalence in pigs, and consequently fewer human infections. For grazing animals such as cattle or small ruminants, it was assumed to be too difficult to implement effective biosecurity [10].

Putting new interventions targeted at toxoplasmosis into practice would be expected to reduce the number of human infections; however, the net benefits for society are unknown. A Cost-Benefit Analysis, also referred to as a Social Cost-Benefit Analysis (SCBA), expresses human health, animal health and costs in comparable terms (i.e. monetary units) and is therefore the methodology to apply when evaluating the net benefits of a new One Health intervention [15, 16]. An SCBA assesses the costs and benefits for a range of social domains and stakeholders and identifies those who benefit and parties that have to pay for the intervention. Here, we describe an SCBA studying two interventions in the food chain to reduce *T*. *gondii* infections in humans in the Netherlands.

## Methods

### Evaluation of intervention measures

The costs and benefits of implementing two interventions were calculated: freezing meat intended for undercooked and raw consumption, and improving biosecurity on pig farms (hereafter called freezing meat intervention and biosecurity intervention, respectively). We assumed that both interventions would be implemented by law, at least at European Union (EU) level, or globally. Following the assumption of large-scale introduction of interventions, trade effects were ignored in the current study.

### Design of the SCBA

**Fig 1:**
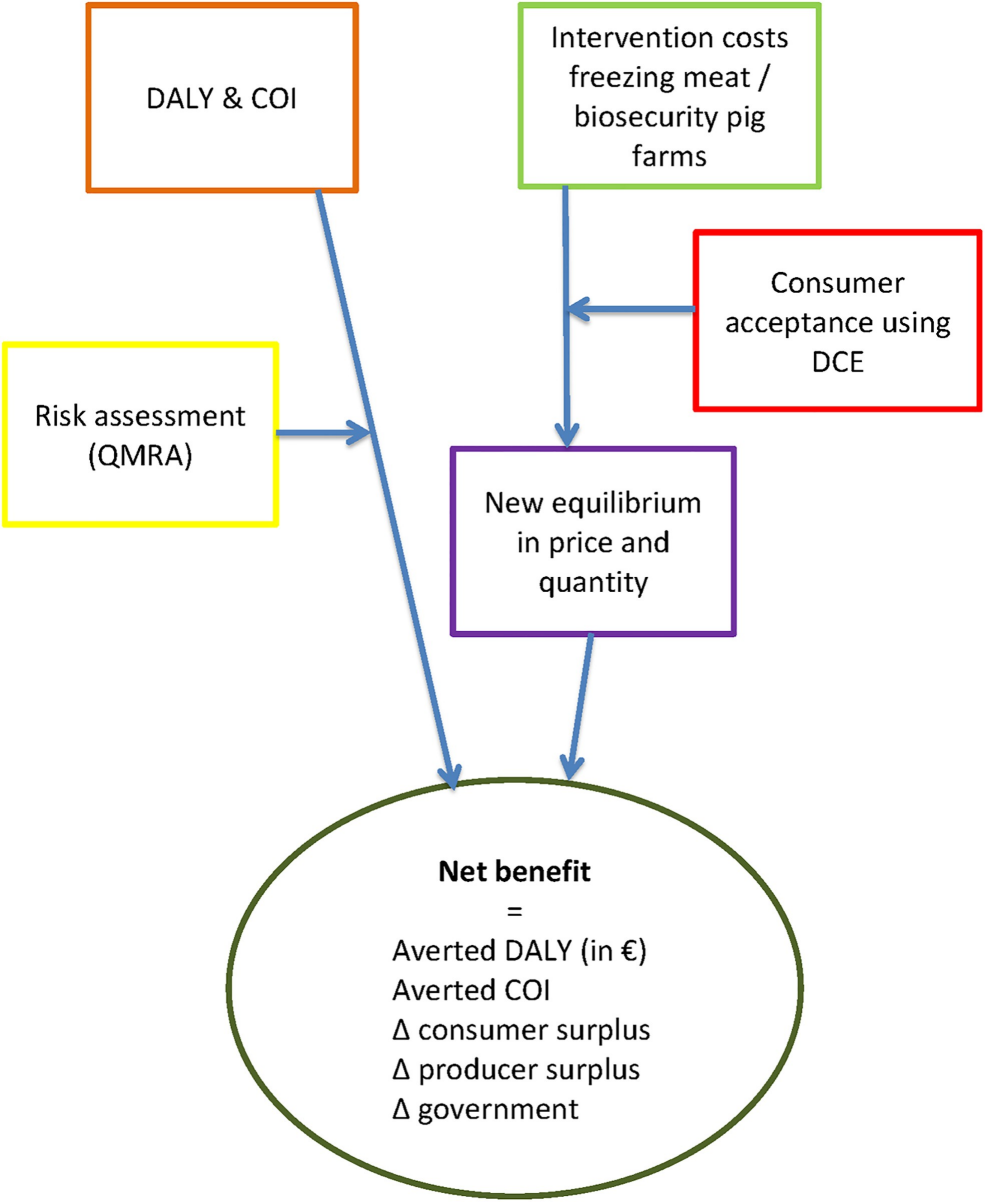
Design of the SCBA. DALY = Disability Adjusted Life Year, COI = cost-of-illness, DCE = Discrete Choice Experiment, QMRA = Quantitative Microbial Risk Assessment.

The design of this SCBA has been described in [16]. In short, several types of input data, partly derived from primary data, partly derived from models, were incorporated in the SCBA, see Fig 1, and are discussed in more detail hereafter. Not all data were available and assumptions had to be made. We defined a ‘minimum scenario’ and a ‘maximum scenario’, using input parameters with least benefits to society and input parameters with most benefits to society, respectively.

### Estimation of incidence, disease burden, cost-of-illness and attribution to meat

At first, we need *T*. *gondii-*related incidence, burden of disease (BoD), and cost-of-illness (COI) estimates. In Northwestern Europe, including the Netherlands, *T*. *gondii* type II predominates [17, 18] and potential virulence differences between types were not taken into account. Using an incidence- and pathogen-based approach, [19] BoD and COI for toxoplasmosis and associated sequelae were estimated for 2016. Incidence, mortality rates and sequelae of congenital toxoplasmosis were estimated based on described methods and data [20, 21] and updated to 2016 using the number of live births as reported by Statistics Netherlands [19–22]. Chorioretinitis, an inflammation of the choroid and retina in the eye, was the only health outcome considered for acquired toxoplasmosis [20]. Infections in immunocompromised patients were not included in the BoD due to data restrictions. Potential associations of toxoplasmosis with psychiatric disorders are debated, however solid links between cause and effects have not been established [23]. Psychiatric disorders because of toxoplasmosis were therefore not considered in the BoD. The outcome tree for congenital *T*. *gondii* infections considers the health outcomes: chorioretinitis, intracranial calcification, hydrocephalus, central nervous system abnormalities, and fetal death [20]. BoD was expressed in Disability Adjusted Life Years (DALY), a metric combining the Years of Life Lost (YLL) due to premature mortality and the Years Lost due to Disability (YLD) [24]. The estimation of the BoD is described in detail in the Supporting Information files (S1 Text).

Healthcare costs, patient costs, special education costs, and productivity losses due to temporary work absences of patients or their caregivers were considered in the COI estimates. These were based on Mangen et al. (2015) and updated to 2016 prices [7] (S1 Text).

Using an expert elicitation study, human *T*. *gondii* cases and associated BoD and COI were attributed to five major exposure pathways (food, environment, direct animal contact, human-human transmission and travel) and eleven food groups [25]. Based on these estimates we calculated the meatborne-attributable fraction of BoD and COI for the year 2016 (see S1 and S2 Tables in the Supporting Information files).

### Quantitative Microbial Risk Assessment (QMRA)

To estimate the reduction in the number of human toxoplasmosis cases following implementation of the two interventions as well as associated BoD and COI, we used output data from an update of the quantitative microbiological risk assessment (QMRA) model for meatborne *T*. *gondii* infections [26] (see S2 Text in the Supporting Information files).

### Interventions

#### Freezing meat intervention

To limit the intervention costs and increase acceptance by consumers, the freezing meat intervention was considered for meat products with a high relative contribution: spicy steak tartare (also known as filet américain, made from beef), beef steak, lamb chop, and leg of mutton [26]. Information from representatives of the Dutch meat industry revealed that 50% of all steak tartare is already produced from meat that was frozen previously. Therefore, for steak tartare, total future freezing costs were calculated for the remaining 50% and effectiveness estimates were limited to the incremental 50% of meat that had to be frozen. The numbers of the specific meat products consumed were retrieved from the Dutch National Food Consumption Survey performed in 2010 [27], see Table 1. Total consumption of all meat products was based on a Dutch report [28]. Additional assumptions are described in S3 Text in the Supporting Information files.

**Table 1:** Input parameters for the economic model.

Description	Point estimator	Unit	Min	Max	Source
**BoD and COI attributable to meatborne toxoplasma infections in 2016**					
DALYs by toxoplasmosis via meatborne infections^a^	326				Estimated*
DALY value	50,000	€			[29]
COI of toxoplasmosis via meatborne infections^a^	7.9	million			Estimated*
**Freezing meat intervention**					
Pork meat consumption^b^	37.4	Kg			[28]
Beef meat consumption^b^	14.2	Kg			[28]
Mutton meat consumption^b^	1.2	Kg			[28]
Steak tartare portion size^c^^d^		g	11	53	[27]
No of portions steak tartare^c^^d^	330	million			[27]
Meat percentage steak tartare/portion^d^		%	50.84	73.96	QMRA
Steak (beef) portion size		g	44	224	[27]
No of steak portions^d^	14	million			[27]
Lamb chop portion size		g	28	214	[27]
No of lamb chop portions^d^	3	million			[27]
Leg of mutton portion size	158	g			[27]
No of leg of mutton portions^d^	0.8	million			[27]
Price elasticity meat	-0.7				[30]
Freezing costs/kg		€	0.10	0.15	^e^
**Biosecurity intervention**					
No of fattening pig farms	4,000				[31]
No fattening pigs/farm	1,450				[31]
No fattening pigs slaughtered/year	15,034,000				[31]
Fattening pigs/lorry when delivered	200				^e^
No of fattening pigs tested/lorry			1	10	^e^
Cost serological test	5	€			[32]
Positive tested farms^f^		%	12	20	[33, 34]
Annual costs rodent control/pig farm		€	400	4000	^g^
Feed cost/fattening pig/year^f^	65	€			[31]
Less spilled feed		%	0	0.1	Assumption
Costs for an additional audit (4 hours)	132	€			[35]^d^
Effectiveness	1	%			[36]

^a^ discounted at 3% a large part of the associated BoD (in particular the sequelae) and costs do not occur in the year of the infection itself, but happen later in life,

^b^per person and year in the Netherlands,

^c^steak tartare also known as filet americain,

^d^consumed in the Netherlands per year,

^e^personal communication VION Food group,

^f^assumption based on quadrupling the % of positive tested farms, based on the % of infected pigs in a recent study which is four times higher than found in a previous study,

^g^personal communication branch organisation of rodent control, averaged 2013–2015,

BoD = Burden of Disease, COI = Cost-of-Illness, DALY = disability adjusted life year,

*We used the average as point estimator, for more details see S1 and S2 Tables.

#### Biosecurity intervention

Controlled housing at pig farms has the potential to prevent *T*. *gondii* infections of pigs. The biosecurity intervention in this study would entail a practical risk-based surveillance program on top of the currently established quality assurance and monitoring at pig farms in the Netherlands. In the case of detection of *T*. *gondii* seropositive pigs during screening at the slaughterhouse, the fattening pig farm would be assumed to conduct additional or intensified on-farm intervention measures to control toxoplasmosis. This would include an additional audit and additional measures to limit exposure to the risk factors identified (see S3 Text for additional information), resulting in additional costs of €400 (minimum) and €4,000 (maximum)/affected farm (Table 1) and an improved rodent control resulting in reduced production costs (Table 1). We assumed that in the minimum scenario in 75% of the farms with seropositive pigs intensified rodent control is necessary; in the maximum scenario, this is assumed to be 25%.

### Discrete Choice Experiment (DCE)

Freezing and thawing of meat will result in safer meat but might impact the physical quality of meat (e.g. moisture loss, and changes in color, and pH) [37–40] potentially influencing consumers’ attitudes. Preference of meat consumers for different attributes of frozen meat were studied in a Discrete Choice Experiment (DCE), (see S4 Text) [41]. As consumers reported a preference for fresh, non-frozen meat in the DCE, the willingness-to-pay was negative (Table 2).

**Table 2:** Attribution to meatborne toxoplasmosis, meat to be frozen, DALYs averted, and WTP for the freezing meat intervention.

	Attribution to meatborne toxoplasmosis (%)	Meat to be frozen (tons)		DALYs averted (N)		WTP/kg
		Min^a^	Max^b^	Min^a^	Max^b^	
Steak tartare	79.82	887.7	6,502.1	208.16	312.25	-€1.64
Beef steak	1.46	6,254.8	32,072.3	3.81	5.71	-€1.13
Lamb chop	0.04	84.7	654.5	0.11	1.16	€0.05
Leg of mutton	3.73	70.8	177.5	9.73	14.60	€0.05
Total	85.05	7298	39406.4	221.81	333.72	

^a^ using least economically favorable input parameters,

^b^ using most economically favorable input parameters,

^c^ preferences for leg of mutton were not assessed by DCE, we assumed the same WTP as for lamb chop, WTP = willingness-to-pay

### Stakeholders

The stakeholders included in this SCBA were consumers (meat consumers, general population at risk for toxoplasmosis), producers (farmers, slaughterhouses, freezing companies, meat processing industry, retailers), and government (including health care and special education). Both interventions would affect consumers: fewer human *T*. *gondii* infections would result in better health (fewer DALYs lost), lower patients’ costs and fewer productivity losses. Due to additional intervention costs, meat prices would rise. The negative WTP estimates in combination with extra costs due to freezing resulted in a double decline in consumers’ demand for these high-risk meat products. The confidence intervals around the WTP estimates comprised the ‘0’, therefore, no change of WTP was considered in the maximum scenario. As consumers’ preferences in experiments may differ from their behavior in daily life [42] only 50% of WTP calculations were taken into account in the minimum scenario. The assessment of the consumer surplus (an economic measure of consumer benefit) is explained in S4 Text.

The interventions will affect the producer surplus, as higher prices of meat will influence consumers’ demand for meat. As we assume perfect competition among freezing meat companies and slaughterhouses, the supply curve is horizontal and there is no difference between market price and supply curve; therefore, we do not take the producer surplus (the benefit for selling the product, see S4 Text for explications) of the freezing meat intervention into account [29]. The same applies to the slaughterhouses in the biosecurity intervention. Since only small changes in the supply of meat throughout the meat chain are expected, we assumed that changes in supplies of meat had no effect on the profit of any of these stakeholders (i.e. slaughterhouses, transport companies, freezing industries, food retailers) in the Netherlands. Additional interventions costs were assumed to be passed through to the consumer, so that welfare changes for these stakeholders were assumed to be zero. Only in case of the biosecurity intervention, affected pig farmers will experience a negative welfare effect. The incurred costs for improving biosecurity cannot be fully compensated by the additional benefits (fewer feed costs) and cannot be passed on to consumers, since they will involve only a selection of all pig farmers.

The final stakeholder is the government who, with decreasing number of *T*. *gondii* infections will incur less healthcare costs and less special education costs. However, when farmers have a lower income due to *T*. *gondii*-related investments, and the freezing meat intervention leads to a lower production of meat, the government will receive less tax revenue. As lower tax revenues will be compensated by consumers in higher taxes elsewhere, we did not include these in our study.

### Economic evaluation

All available input for the SCBA (see Tables 1 and 2) was synthesized using a Microsoft Excel model. For the reference scenario, we assumed existing preventive measures by means of educating pregnant women, to be unchanged and no changes in incidence or prevalence of *T*. *gondii* infections over time. The model estimates the net value by comparing the reference scenario with the two alternative scenarios including reduced *T*. *gondii* transmission by calculating the difference between the costs and benefits in the alternative and the reference scenario. The net values of costs and benefits are presented per intervention per year, per stakeholder, using DALY and COI estimates. The monetary value of a DALY was assumed to be €50,000 [29]. All costs were expressed for the year 2016, and we indexed price levels using Dutch consumer price indices as provided by Statistics Netherlands.

### Sensitivity and scenario analyses

For both interventions, we performed different scenario analyses. We varied the baseline value of a DALY from €50,000 (baseline) to € 100,000/DALY for both interventions [29].

For the freezing meat intervention, we calculated results for not assuming perfect competition between the freezing companies and in this case including the producer surplus in the model. We varied the meatborne attribution (44% of all toxoplasmosis infections in the baseline analysis) with a 50% higher and 50% lower estimate and we varied the annual BoD (expressed as DALYs/year) and COI in the population using the 2.5% and 97.5% (748 DALY/year and €18.3 million/year in baseline versus 506 DALY/year and €5.1 million/year (2.5%) and 1063 DALY/year and €53.2 million/year (97.5%), see S1 Table. Lastly, we combined the 50% higher meatborne attribution estimate with a scenario of 1063 DALYs/year and €53.2 million/year.

For the biosecurity intervention, we changed the attribution of pig meat products to toxoplasmosis as provided by the QMRA with attribution of pig meat products based on expert elicitations (66% versus 14% in the baseline). In addition, because the effects and the endurance of effects in the biosecurity measure are mostly unknown, we varied effectiveness in additional sensitivity analyses to 10% effect (versus 1% in baseline), and endurance of 5 and 10 years were used (versus 1 year in baseline), whereby using a 3% discount rate [29].

## Results

### Freezing meat products

The products steak tartare, lamb chops, leg of mutton, and beef steaks are considered to attribute a risk for meatborne *T*. *gondii* infection but no pork products according to the QMRA and the amount of these meat products to be frozen is shown in Table 2. The relative contribution of all pork meat is 12%. Depending on the assumptions on portion sizes this varies from 7,298 tonnes up to 39,406.4 tonnes for all four products annually for the Netherlands. QMRA results show that freezing these meat products would lead to a decline of 85% of all meatborne *T*. *gondii* infections and its corresponding DALYs, ranging from 222 to 334 DALYs averted.

Table 3 presents costs and benefits for freezing the four meat products from Table 2. Using the least economically favorable input parameters in the model (the ‘minimum scenario’) the intervention would lead to annual net benefit of €10.6 million and €0.6 million for respectively steak tartare and leg of mutton, but would not render benefits for the other two products. Using the most favorable input parameters (the ‘maximum scenario’) freezing steak tartare and leg of mutton would lead to annual total net benefits of €31 million and €1.5 million respectively. Freezing the other risk meat products would still lead to net costs for society. Monetized DALYs contribute most to benefits of the freezing intervention, followed by avoided healthcare costs (Table 3). Freezing costs are lowest for leg of mutton (€0.008 to €0.028 million) and highest for beef steak (€0.6 to €4.8 million) in line with the volume consumed in the Netherlands. Freezing beef steak would result in the lowest consumer surplus (ranging from -€0.6 million to -€2.7 million).

**Table 3:** Net benefits for the stakeholders involved with the freezing meat interventions in 2016 (€)*1000.

	Steak tartare		Beef steak		Lamb chop		Leg of mutton	
Stakeholders^a^	Min	Max	Min	Max	min	max	min	max
Freezing companies^b^	-975	-89	-4,811	-626	-98	-8	-28	-8
	+975	+89	+4,811	+626	+98	+8	+28	+8
Consumers								
Freezing costs	-975	-89	-4,811	-626	-98	-8	-28	-8
DALYs averted	10,408	15,612	190	286	5.3	8	487	730
Patient costs	12	24	0.2	0.4	0.0	0.0	0.6	1.1
Productivity losses	199	362	3.6	6.6	0.1	0.2	9	17
Consumer surplus	-907	-112	-2,722	-622	-10	-8	-4	-3
Government								
Healthcare costs	1,836	15,136	33.6	277	0.9	7.8	86	708
Special education costs	3.2	143.3	0.06	2.6	0.0	0.1	0.2	6.7
**Net benefits**^c^	**10,576**	**31,077**	**-7305**	**-625**	**-102**	**-0.6**	**550**	**1,452**

Min: using input parameters that result in economically least favorable outcomes, Max: using input parameters that result in economically most favorable outcomes,

^a^ we assumed no change in costs for farmers and retailers

^b^ Intervention costs occurring in freezing companies will be put through to consumer (so at slaughterhouse level it will be zero),

^c^ note: a negative number corresponds with costs, a positive number with savings

### Improving biosecurity on pig farms

Improving biosecurity on pig farms results in costs ranging from €1.1 million in the ‘maximum scenario’ to €2.5 million in the ‘minimum scenario’ as costs easily outweigh the benefits of the intervention (Table 4). In both scenarios, farmers have the highest costs by implementing professional rodent control of mice and casts from the stables. In addition, consumers incur costs as serological costs paid by slaughterhouses would be put through to consumers; leading to higher consumer prices. Most benefits are realized by monetized DALYs, ranging from 47 to 31 DALYs averted, and from avoided healthcare costs.

**Table 4:** Net benefits for the stakeholders involved with the biosecurity interventions in 2016 (€)*1000.

	Biosecurity intervention	
Stakeholders	Min	Max
Producers		
Farmers	-2,103	-701
Slaughterhouses^a^	-439	-482
	+439	+482
Consumers		
Intervention costs slaughterhouses	-439	-482
DALYs averted	16	23
Patient costs	0.0	0.0
Productivity losses	0.3	0.5
Government		
Healthcare costs	3	23
Special education costs	0.0	0.2
**Net benefits**^b^	**-2,525**	**-1,136**

Min: using least economically favorable input parameters, Max: using most economically favorable input parameters,

^a^Intervention costs occurring in slaughterhouses will be put through to consumer (so at slaughterhouse level it will be zero),

^b^ note: a negative number corresponds with costs, a positive number with savings

### Sensitivity analyses

Results of the sensitivity and scenario analyses are presented in Table 5. In all cases, freezing steak tartare and leg of mutton would still lead to savings to society. Increasing the DALY value to €100,000 had the highest impact on results making freezing both meat products even more favorable. Increasing the meatborne attribution estimate with 50% in combination with a higher annual burden (expressed as higher number of DALYs/year and COI/year) also resulted in more net benefits to society. If no perfect competition between freezing companies was assumed, this had hardly any effect on the results.

A higher DALY value and increasing the effectiveness of the biosecurity intervention to 10% instead of 1% would not result in net benefits for society. In addition, when an attribution of pig meat products to toxoplasmosis was based on expert elicitations (66%) instead of the pig meat attribution from the QMRA of 12%, it did not result in net benefits for society (Table 5). Results for higher effectivity implying a fivefold (5%) or a tenfold (10%) reduction of the disease burden of toxoplasmosis associated with the biosecurity intervention lasting to pork for five or ten years are presented in S1–S6 Figs.

**Table 5:** Results sensitivity and scenario analysis, net benefits in €1000.

Freezing meet intervention				
	Min	Relative change compared to main analysis %	Max	Relative change compared to main analysis %
**Main analysis**				
Steak tartare	10,576		31,077	
Beef steak	-7,305		-676	
Lamb chop	-102		-0.6	
Leg of mutton	551		1,452	
**DALY valuation €100,000**				
Steak tartare	20,984	98	46,689	50
Beef steak	-7,114	-3	-390	-42
Lamb chop	-96	-5	7	-1360
Leg of mutton	1,037	88	2,182	50
**No perfect competition between freezing companies**				
Steak tartare	10,495	-1	31,055	0
Beef steak	-7,526	3	-800	18
Lamb chop	-104	2	-2.6	339
Leg of mutton	550	0	1,451	0
**Meatborne attribution 50% lower (22% versus 44% in baseline)**				
Steak tartare	9,551	-10	23,244	-25
Beef steak	-7,323	0	-819	21
Lamb chop	- 102	1	-5	683
Leg of mutton	503	-9	1,086	-25
**Meatborne attribution 50% higher (66% versus 44% in baseline)**				
Steak tartare	11,601	10	38,910	25
Beef steak	-7,286	0	-532	-21
Lamb chop	-101	-1	3	-683
Leg of mutton	598	9	1,818	25
**Lower estimate (2.5%) for BoD and COI in the population**				
Steak tartare	6,525	-38	20,907	-33
Beef steak	-7,379	1	-862	28
Lamb chop	-104	2	-6	886
Leg of mutton	361	-34	976	-33
**Higher estimate (97.5%) for BoD and COI in the population**				
Steak tartare	15,773	49	44,125	42
Beef steak	-7,210	-1	-437	-35
Lamb chop	-99	-3	6	-1137
Leg of mutton	794	44	2.,062	42
**Meatborne attribution 50% higher & higher estimate (97.5%) for BoD and COI in the population**				
Steak tartare	17,225	63	55,226	78
Beef steak	-7,183	-2	-234	-65
Lamb chop	- 98	-3	12	-2104
Leg of mutton	861	56	2,581	1725
**Biosecurity intervention**^a^				
Main analysis	-2,525		-1,136	
DALY valuation €100,000	-2,509	-1	-1,113	-2
Effectiveness 10%	-2,362	-7	-716	-37
Attribution of pig meat products based on expert elicitations	-2,469	-2	-1,010	-11

^a^ additional sensitivity analyses are presented in figures in the Supporting Information files,

BoD = Burden of Disease, COI = Cost-of-illness

## Discussion

In this SCBA we compared the costs and benefits of adding two interventions targeted at reducing the *T*. *gondii*-related BoD to current practice which is focused solely on educating pregnant women and other risk groups. The intervention related to freezing high-risk meat (products) is far more effective in reducing BoD than the intervention to improve the biosecurity on pig farms. Freezing steak tartare and mutton leg yield annual net social benefits in both the minimum and maximum scenario, ranging from €11 million to €31 million for steak tartare and €0.6 to €1.5 million for leg of mutton. These results remained robust in sensitivity analysis. The estimated risk of infection per portion of steak tartare is low (4.5 × 10^−4^) but this product is eaten frequently in the Netherlands. Leg of mutton is eaten infrequently in the Netherlands, but in this case the risk of infection per portion is high (1.1 × 10^−2^) due to the high prevalence and bradyzoite concentration in sheep and the heating distribution that allows for the possibility of undercooking.

The DCE performed in this study revealed that consumers do not prefer to buy industrially frozen (and thawed) meat [41]. Additional information for consumers seems necessary to convince them to buy ‘toxoplasma-safe’ meat. On the other hand, half of the meat intended for producing steak tartare and similar meat products is currently frozen, and consumers do not seem to have knowledge on this fact nor notice a difference between the two variants of the product. The intervention related to improve biosecurity on pig farms would result in net costs in all scenarios ranging from €1 to €2.5 annually. These results are driven by high costs for farmers and consumers and by an assumed 1% effectivity of the intervention with DALYs averted and associated benefits being low. This could be influenced by the already high level of biosecurity on pig farms in the Netherlands since the larger part of pig production is under controlled housing conditions [43].

Although vaccination of the cat population was identified as being a potentially effective intervention to prevent humans to get infected with oocysts [16], there is yet no vaccine available and the feasibility of implementing such an intervention is questionable (See S5 Text, Bonacic Marinovic et al. submitted), therefore cat vaccination was not further studied in the current SCBA.

This SCBA reveals several strengths and limitations. As far as we know, only Van Asseldonk et al evaluated the costs and benefits of an intervention for toxoplasmosis by improving biosecurity at pig farms, considering relevant stakeholders [44].

Strengths of the current study are the incorporation of updated information of attributing risk meat products for acquiring toxoplasmosis and the assessment of consumers’ preferences towards frozen meat. The main limitations of the study are the uncertainty of incidence of *T*. *gondii* infections, for both congenital as acquired infections, and the corresponding BoD and COI, the uncertainty of the attribution to the main pathways of toxoplasmosis by expert elicitation and to the meat products by QMRA, and the lack of effectiveness data applied to the biosecurity intervention. Currently, we assumed effectiveness of 1%. If the effectiveness turns out to be more than 1%, that would lead to a higher net value for the biosecurity intervention in this SCBA. The intervention, therefore, might be more favorable than we can conclude from our best estimates, although the scenario analysis indicates that increasing biosecurity would still result in net costs at 10% effectivity. By freezing and thawing meat, additional steps are introduced in the meat chain, with a risk of introducing additional hazards with a negative impact on human health. On the other hand, freezing may also negatively impact the viability of other foodborne pathogens such as *Campylobacter* [45]. These possibilities were not considered in the current study.

A main assumption was that the interventions would be imposed by law in the European Union (at the least), and that trade/market distortion could be ignored. However, if this assumption does not hold, our results are no longer valid. In the Netherlands around 75% of all meat is exported, mostly to EU countries [46].

Regarding congenital infections, we only included productivity losses for caregivers, not for children born with these complications, based on a French study [47]. In France, contrary to the Netherlands, screening during pregnancy is implemented, with possibly a different population of congenital *T*. *gondii* cases. Furthermore, DALY estimations were based on fetal losses from only 24^th^ week of gestation and onwards. Finally, productivity losses for fetal and neonatal deaths were excluded from our calculation based on Dutch SCBA guidance [29]. This differs from other cost-benefit analyses (e.g. [48]) and other SCBA guidelines.

Price elasticity of meat was not specifically targeted at the separate meat products, and consumers shifting to consuming other meat products with a different predicted probability of causing *T*. *gondii* infection [49, 50] were disregarded.

To evaluate the full economic impact of interventions to control a zoonosis, the influence on the human and animal health sector need to be integrated for decision makers in all sectors [15, 51]. We performed an SCBA to assess interventions targeted at the prevention of toxoplasmosis and gained insight in a range of mechanisms that influenced total net monetary results. Using conservative input parameters for the biosecurity intervention in the SCBA model, the intervention would result in high net costs for farmers and consumers, with only limited positive effects for consumers. On the contrary, freezing high risk meat products would result in net benefits for society. More specifically, freezing all steak tartare instead of the current 50% and leg of mutton are expected to be efficient options to reduce the burden of toxoplasmosis and increase food safety.

## Supporting information

S1 Table Attribution of toxoplasmosis to main pathways. (DOCX)

S2 Table Annual average costs for patients, healthcare, special education, and productivity losses due to *T*. *gondii* infection in the Netherlands, 2016. (DOCX)

S1 Text Incidences, Burden of disease (BoD) and cost-of-illness (COI). (DOCX)

S2 Text Quantitative Microbial Risk Assessment (QMRA). (DOCX)

S3 Text Assumptions regarding the interventions. (DOCX)

S4 Text Glossary. (DOCX)

S5 Text Cat vaccination. (DOCX)

S1 Fig Assuming effectiveness 1%, lasting for 5 years in the minimum scenario. (PDF)

S2 Fig Assuming effectiveness 1%, lasting for 5 years in the maximum scenario. (PDF)

S3 Fig Assuming effectiveness 1%, lasting for 10 years in the minimum scenario. (PDF)

S4 Fig Assuming effectiveness 1%, lasting for 10 years in the maximum scenario. (PDF)

S5 Fig Assuming effectiveness 10%, lasting for 10 years in the minimum scenario. (PDF)

S6 Fig Assuming effectiveness 10%, lasting for 10 years in the maximum scenario. (PDF)
