# Supplementary material for: A social cost-benefit analysis of two One Health interventions to prevent toxoplasmosis
Source: PLoS One. 2019 May 10;14(5):e0216615. doi: 10.1371/journal.pone.0216615 (PMC6510435; doi:10.1371/journal.pone.0216615)
Supplement: S1 Table — (DOCX) [file pone.0216615.s001.docx]

**S1 Table. Attribution of toxoplasmosis to main pathways in the Netherlands, 2016 – Average and 95% uncertainty intervals in brackets^a^**

|  | **Food** | **Whereof meatborne** | **Environment** | **Human** | **Animal** | **Travel- related^b^** | **Total** |
| --- | --- | --- | --- | --- | --- | --- | --- |
| Incidence | 428  (256-638) | **334**  (200-497) | 278  (166-414) | 6.9  (4-10) | 19  (11-29) | 35  (21-53) | 767  (459-1143) |
| Deaths | 6.7  (4.3-11) | **5.3**  (3.3-8.3) | 4.4  (2.8-6.9) | 0.1  (0.1-0.2) | 0.3  (0.2-0.5) | 0.6  (0.4-0.9) | 12.1  (8-19) |
| DALY per year |  |  |  |  |  |  |  |
| undiscounted (0%) | 1,062  (711-1530) | **827**  (554-1192) | 689  (462-992) | 17  (11-25) | 48  (32-69) | 88  (59-126) | 1,902  (1275-2741)) |
| discounted (3%) | 418  (282-560) | **326**  (220-462) | 271  (183-385) | 6.7  (4.6-9.6) | 19  (13-27) | 34  (23-49) | 748  (506-1063) |
| Cost-of-illness^c^ | 10.2  (2.8-29.7) | **7.9**  (2.2-23.1) | 6.6  (1.8-19.3) | 0.2  (0.05-0.5) | 0.5  (0.1-1.3) | 0.8  (0.2-2.4) | 18.3  (5.1-53.2) |

This table is adapted from Mangen et al [1]. More details on incidence, deaths, DALYs and COI, and the underlying assumptions and uncertainty ranges are presented in Mangen et al. [1]. Note they used a 1.5% discount rate for DALYs and 4% for costs.

^a^ Values represents the uncertainty w.r.t. incidence estimates and health outcomes, but not the uncertainty w.r.t. attribution, here we had used the most likely values as estimated by experts (for details see Havelaar et al [2].

^b^ Travel-related infections were acquired outside the Netherlands by one of the four major route of transmissions, i.e. food, environment, direct animal contact and human-human transmission.

^c^ M€ per year, discounted at 3% and expressed in 2016 euros

**References**

1. Mangen MJ, Friesema IHM, Haagsma JA, van Pelt W. Disease burden of food-related pathogens in the Netherlands, 2016. Bilthoven: RIVM, 2017 Contract No.: RIVM report nr. 2017-0097.

2. Havelaar AH, Galindo AV, Kurowicka D, Cooke RM. Attribution of foodborne pathogens using structured expert elicitation. Foodborne pathogens and disease. 2008;5(5):649-59. Epub 2008/08/09. doi: 10.1089/fpd.2008.0115. PubMed PMID: 18687052.
