# Supplementary material for: A social cost-benefit analysis of two One Health interventions to prevent toxoplasmosis
Source: PLoS One. 2019 May 10;14(5):e0216615. doi: 10.1371/journal.pone.0216615 (PMC6510435; doi:10.1371/journal.pone.0216615)
Supplement: S2 Table — (DOCX) [file pone.0216615.s002.docx]

**Table S2. Annual average costs for patients, healthcare, special education, and productivity losses due to *T. gondii* infection in the Netherlands, 2016 (*1000 in 2016 euros)^a^**

|  | **Patients** | | | **Healthcare** | | **Special education** | | **Productivity losses** | |
| --- | --- | --- | --- | --- | --- | --- | --- | --- | --- |
| **Undiscounted** |  | | |  | |  | |  | |
| **Beef and lamb** | 11 | | | 5480 | | 33 | | 174 | |
| **Pork** | 25 | | | 12012 | | 71 | | 382 | |
| **Poultry** | 2 | | | 1149 | | 7 | | 36 | |
| **Dairy** | 2 | | | 1101 | | 7 | | 35 | |
| **Fish & shellfish** | 2 | | | 885 | | 5 | | 28 | |
| **Produce** | 3 | | | 1388 | | 8 | | 44 | |
| **Other foods** | 1 | | | 550 | | 3 | | 17 | |
| **Humans and animals** | 3 | | | 1364 | | 8 | | 43 | |
| **Total** | 49 | | | 23929 | | 142 | | 760 | |
| **Discounted at 3%** | |  |  | |  | |  | |  |
| **Beef & Lamb** | | 11 | 2202 | | 24 | | 104 | |  |
| **Pork** | | 25 | 4828 | | 25 | | 228 | |  |
| **Poultry** | | 2 | 462 | | 5 | | 22 | |  |
| **Dairy** | | 2 | 442 | | 5 | | 21 | |  |
| **Fish & shellfish** | | 2 | 356 | | 4 | | 17 | |  |
| **Produce** | | 3 | 558 | | 6 | | 26 | |  |
| **Other foods** | | 1 | 221 | | 2 | | 10 | |  |
| **Humans and animals** | | 3 | 548 | | 6 | | 26 | |  |
| **Total** | | 49 | 9617 | | 103 | | 454 | |  |

^a^ The reference scenario in the SCBA, This table is adapted from Mangen et al [1]. These authors estimated the costs for the year using a 0% and 4% discount rate. For more details and the uncertainty range, see Mangen et al. [1]

**References**

1. Mangen MJ, Friesema IHM, Haagsma JA, van Pelt W. Disease burden of food-related pathogens in the Netherlands, 2016. Bilthoven: RIVM, 2017 Contract No.: RIVM report nr. 2017-0097.
