# Supplementary material for: A social cost-benefit analysis of two One Health interventions to prevent toxoplasmosis
Source: PLoS One. 2019 May 10;14(5):e0216615. doi: 10.1371/journal.pone.0216615 (PMC6510435; doi:10.1371/journal.pone.0216615)
Supplement: S1 Text — (DOCX) [file pone.0216615.s003.docx]

**S1 Text**

**1. BoD methods**

BoD was expressed in Disability Adjusted Life Years (DALY), a metric combining the Years of Life Lost (YLL) due to premature mortality and the Years Lost due to Disability (YLD) [1]. YLL is calculated by multiplying the number of deaths due to congenital toxoplasmosis with their remaining life expectancies in years (i.e. 85.68 years [2]), whereby only fetal losses starting from 24 weeks of gestation were taken into account [3]. YLD was derived by accumulating over all cases and all health outcomes the product of the duration of the illness and the disability weight of a specific disease (scale ranging from 0 (perfect health) to 1 (dead)). We used the DALY estimation recently updated with European disability weights [4], for more details see Mangen et al [5, 6]. Non-fatal symptomatic *T. gondii* infections resulted in life-long remaining symptoms [3]. We assumed that the monetary value of a DALY corresponds with the monetary value of the Quality Adjusted Life Year (QALY) with a Dutch standard value of €50.000 [7].

**2. COI calculations**

Acquired and congenital chorioretinitis was assumed to never result in blindness in both eyes [8, 9]. Blindness in one eye, however, seldom results in work inability, and these costs were disregarded. The long-term implications of non-fatal congenital toxoplasmosis with respect to special education and temporary productivity losses of caregivers were included. In addition, therapeutic help (between age 2 and 7 years), allowing them to catch up with their peers and neurological examination up to 20 years of age was also included [10, 11]. Following Berrébi et al. we assumed that academic and cognitive development in children with a chronic manifestation of toxoplasmosis would be the same as that of non-infected children at adulthood [12], and therefore the long-term production losses of these children were assumed to be negligible. Finally, we did not include production losses associated with premature mortality as recommended by the Dutch guidance on SCBA [7].

Contrary to earlier estimates [5] we used the newly available reference prices for health economic evaluations from the Dutch healthcare institute [13]. In order to better reflect the current situation of the labour market, and following the most recent guideline, the friction period (i.e. the period needed to replace a sick, deceased worker) was changed from 23 weeks to 12 weeks [13].

**References**

1. Murray CJ. Quantifying the burden of disease: the technical basis for disability-adjusted life years. Bulletin of the World Health Organization. 1994;72(3):429-45. Epub 1994/01/01. PubMed PMID: 8062401; PubMed Central PMCID: PMCPMC2486718.

2. WHO. WHO methods and data sources for global burden of disease estimates 2000-2011. Geneva: WHO, 2013.

3. Havelaar AH, Kemmeren JM, Kortbeek LM. Disease burden of congenital toxoplasmosis. Clinical infectious diseases : an official publication of the Infectious Diseases Society of America. 2007;44(11):1467-74. Epub 2007/05/08. doi: 10.1086/517511. PubMed PMID: 17479945.

4. Haagsma JA, Maertens de Noordhout C, Polinder S, Vos T, Havelaar AH, Cassini A, et al. Assessing disability weights based on the responses of 30,660 people from four European countries. Population health metrics. 2015;13:10. Epub 2016/01/19. doi: 10.1186/s12963-015-0042-4. PubMed PMID: 26778920; PubMed Central PMCID: PMCPMC4715333.

5. Mangen MJ, Bouwknegt M, Friesema IH, Haagsma JA, Kortbeek LM, Tariq L, et al. Cost-of-illness and disease burden of food-related pathogens in the Netherlands, 2011. International journal of food microbiology. 2015;196:84-93. Epub 2014/12/22. doi: 10.1016/j.ijfoodmicro.2014.11.022. PubMed PMID: 25528537.

6. Mangen MJ, Friesema IHM, Haagsma JA, van Pelt W. Disease burden of food-related pathogens in the Netherlands, 2016. Bilthoven: RIVM, 2017 Contract No.: RIVM report nr. 2017-0097.

7. Koopmans C, Heyma A, Hof B, Imandt M, Kok L, Pomp M. Werkwijzer voor kosten-batenanalyse in het sociale domein. Amsterdam: SEO Economisch Onderzoek, 2016.

8. Bosch-Driessen LE, Berendschot TT, Ongkosuwito JV, Rothova A. Ocular toxoplasmosis: clinical features and prognosis of 154 patients. Ophthalmology. 2002;109(5):869-78. Epub 2002/05/03. PubMed PMID: 11986090.

9. Faucher B, Garcia-Meric P, Franck J, Minodier P, Francois P, Gonnet S, et al. Long-term ocular outcome in congenital toxoplasmosis: a prospective cohort of treated children. The Journal of infection. 2012;64(1):104-9. Epub 2011/11/05. doi: 10.1016/j.jinf.2011.10.008. PubMed PMID: 22051915.

10. Roser D, Nielsen HV, Petersen E, Saugmann-Jensen P, Norgaard-Pedersen B. Congenital toxoplasmosis--a report on the Danish neonatal screening programme 1999-2007. Journal of inherited metabolic disease. 2010;33(Suppl 2):S241-7. Epub 2010/06/30. doi: 10.1007/s10545-010-9124-4. PubMed PMID: 20585987.

11. Schmidt DR, Hogh B, Andersen O, Fuchs J, Fledelius H, Petersen E. The national neonatal screening programme for congenital toxoplasmosis in Denmark: results from the initial four years, 1999-2002. Archives of disease in childhood. 2006;91(8):661-5. Epub 2006/07/25. doi: 10.1136/adc.2004.066514. PubMed PMID: 16861484; PubMed Central PMCID: PMCPMC2083029.

12. Berrebi A, Assouline C, Bessieres MH, Lathiere M, Cassaing S, Minville V, et al. Long-term outcome of children with congenital toxoplasmosis. American journal of obstetrics and gynecology. 2010;203(6):552.e1-6. Epub 2010/07/17. doi: 10.1016/j.ajog.2010.06.002. PubMed PMID: 20633868.

13. ZIN. Kostenhandleiding: Methodologie van kostenonderzoek en referentieprijzen voor economische evaluaties in de gezondheidszorg. Amsterdam: Zorginstituut Nederland (ZIN), 2015.
