# Supplementary material for: A social cost-benefit analysis of two One Health interventions to prevent toxoplasmosis
Source: PLoS One. 2019 May 10;14(5):e0216615. doi: 10.1371/journal.pone.0216615 (PMC6510435; doi:10.1371/journal.pone.0216615)
Supplement: S2 Text — (DOCX) [file pone.0216615.s004.docx]

**S2 Text**

**Quantitative Microbial Risk Assessment (QMRA)**

The Quantitative Microbial Risk Assessment (QMRA) model calculates the predicted number of human *T. gondii* infections and the relative attribution of different meat products using meatborne exposure data including food consumption data of the Netherlands [1]. The previously published QMRA for toxoplasmosis [2] was updated with:

- information from the Dutch National Food Consumption Survey in 2007-2010 [1],
- with a lower bradyzoite concentration of *T. gondii* for beef products,
- and a new estimate for the effect of salting on *T. gondii* viability

and are described in detail in Deng et al. (in preparation).

The results of the updated QMRA model were used to simulate the number of meatborne *T. gondii* infections for the current situation as well as after implementation of the two interventions. The relative attributions to the total number of predicted infections in the QMRA, were used to attribute the total meatborne BoD and COI to specific meat products.

**References**

1. van Rossum CTM, Fransen HR, Verkaik-Kloosterman J, Buurma-Rethans EJM, Ocké MC. Dutch National Food Consumption Survey 2007-2010 Bilthoven: RIVM; 2011 [2019-3-01]. Available from: <https://www.rivm.nl/bibliotheek/rapporten/350050006.pdf>.

2. Opsteegh M, Prickaerts S, Frankena K, Evers EG. A quantitative microbial risk assessment for meatborne Toxoplasma gondii infection in The Netherlands. International journal of food microbiology. 2011;150(2-3):103-14. Epub 2011/08/26. doi: 10.1016/j.ijfoodmicro.2011.07.022. PubMed PMID: 21864927.
