# Supplementary material for: A social cost-benefit analysis of two One Health interventions to prevent toxoplasmosis
Source: PLoS One. 2019 May 10;14(5):e0216615. doi: 10.1371/journal.pone.0216615 (PMC6510435; doi:10.1371/journal.pone.0216615)
Supplement: S3 Text — (DOCX) [file pone.0216615.s005.docx]

**S3 Text**

**Assumptions regarding the interventions**

**1. Freezing meat intervention**

According to several people from the Dutch meat industry who were interviewed, 50% of all steak tartare is currently already produced from meat that was frozen previously. Therefore, for steak tartare, total future freezing costs were calculated for the remaining 50%. Total capacity of freezing companies for freezing meat in the Netherlands was assessed at 4,500,000 m^3^ (Davey Gerlings, Nekovri, personal communication 9-28-2017). Given the low quantity of meat to be frozen (45-252 m^3^/day) we assumed that an investment neither in new freezing installations, nor in transport facilities would be required. We further assumed that both freezing and transport companies would realize the same profit independent of the type of good that is being frozen or transported. Freezing and thawing was assumed to be implemented at the freezing company, with partly additional transport from slaughterhouse to freezing company. Additional freezing and transport costs were estimated to be €0.10-€0.15 per kg (Table 1). Presuming that the intervention would be implemented by law, the intervention costs were assumed to be passed through to the consumer in the form of a higher consumer meat price.

**2. Biosecurity intervention**

In the Netherlands, working procedures including quality assurance and monitoring to improve food safety are already established at pig farms[1]. The biosecurity intervention in this study entails a practical risk- based surveillance program on top on the currently established quality assurance and monitoring at pig farms in the Netherlands. When *T. gondii* seropositive pigs are detected during screening at the slaughterhouse, the fattening farm is assumed to conduct additional or intensified on-farm intervention measures to control toxoplasmosis. This includes an additional audit for the presence of risk factors and recommendations to the pig farmer how to limit exposure to these risk factors. Interventions on pigs farms such as additional covering of feeders and storage of feed; extra measures to control rodents and extra measures to exclude cats from the stables were taken into account in our study [2]. All these measures resulted in additional costs to be incurred by the affected farmers (€400 (minimum) and €4000 (maximum) based on information from the branch organisation of rodent control, Table 1. As *T. gondii* infections are usually asymptomatic in pigs [3] the intervention was expected not to improve animal health, growth or reproduction figures. However, improved rodent control was assumed to result in reduced feed spillage (0% (minimum) and 0.1% (maximum)), and consequently reduced production costs for the farmer concerned (Table 1).

The number of farms (4000) and pigs (around 15 million) were taken from agrimatie.nl [4], the number of infected pigs (2%) and farms (16%) was based on the literature [5, 6] (Table 1). In the absence of scientific evidence on the effectiveness of existing biosecurity interventions for *T. gondii* prevalence on pig farms, we assumed, similar to Mangen et al., a conservative effectivity of the intervention of 1% [7]. That is, in 1% of the infected and detected farms, the intervention will eliminate *T. gondii*, and the farms remain *T. gondii* free for the duration of 1 year. Serology costs (Table 1) are paid by the slaughterhouses and were assumed to be passed through to the consumer as a higher meat price.

**References**

1. IKB_varken [cited 2017]. Available from: <www.ikbvarken.nl>.

2. EFSA. Technical specifications on harmonised epidemiological indicators for public health hazards to be covered by meat inspection of swine. EFSA J. 2011;9(10):2371.

3. Dubey JP. Toxoplasmosis in pigs--the last 20 years. Veterinary parasitology. 2009;164(2-4):89-103. Epub 2009/06/30. doi: 10.1016/j.vetpar.2009.05.018. PubMed PMID: 19559531.

4. Agrimatie. Informatie over de agrosector. Available from: <www.agrimatie.nl>.

5. van der Giessen J, Fonville M, Bouwknegt M, Langelaar M, Vollema A. Seroprevalence of Trichinella spiralis and Toxoplasma gondii in pigs from different housing systems in The Netherlands. Veterinary parasitology. 2007;148(3-4):371-4. Epub 2007/07/25. doi: 10.1016/j.vetpar.2007.06.009. PubMed PMID: 17646053.

6. Swanenburg M, Boender GJ, Heres L, Koeijer A, Wisselink HJ. Toxoplasma prevalence in Dutch slaughter pigs in the period 2012-2014. In: Vieria-Pinto, editor. Epidemiology and control of hazards in pork production chain - SAFEPORK One health approach under a concept of farm to fork. Porto, Portugal2015. p. 69-72.

7. Mangen MJ, Havelaar AH, Poppe KP, de Wit GA. Cost-utility analysis to control Campylobacter on chicken meat: dealing with data limitations. Risk analysis : an official publication of the Society for Risk Analysis. 2007;27(4):815-30. Epub 2007/10/26. doi: 10.1111/j.1539-6924.2007.00925.x. PubMed PMID: 17958494.
