# Supplementary material for: A social cost-benefit analysis of two One Health interventions to prevent toxoplasmosis
Source: PLoS One. 2019 May 10;14(5):e0216615. doi: 10.1371/journal.pone.0216615 (PMC6510435; doi:10.1371/journal.pone.0216615)
Supplement: S4 Text — (DOCX) [file pone.0216615.s006.docx]

**S4 Text**

**Glossary**

**1. Discrete Choice Experiment (DCE)**

A DCE is a stated preference survey technique that enables the calculation of Willingness-to-Pay (WTP) for different aspects of frozen meat, such as food safety, expiration date, quality of meat, production method, and price [1]. In a DCE-model, the probability that a person chooses a particular alternative is estimated. Consumers’ preferences are provoked by quantifying the relative importance of freezing and other characteristics of consuming risk meat products. Therefore, 671 (response=75%) respondents participating in a panel study were invited to complete a set of online tasks by choosing between two or more scenarios when buying the toxoplasma-related risk meat products identified in this SCBA: beef steak, lamb chop, and steak tartare [2]. The description of the meat product in these tasks was based on its characteristics or ‘attributes’, i.e. price, type of production, expiration date, quality, and either or not freezing during production. The results indicate to which extent the values of the levels of the attributes determine the preference of respondents for a certain scenario. By dividing the coefficients of the attribute that expressed the meat to be frozen by the price parameter, the change in WTP between frozen and unfrozen meat was estimated. The WTP was estimated for certain classes of consumers. For the SCBA we used the weighted mean of the WTP estimates.

**2. Consumer surplus**

We assessed the consumer surplus of freezing meat, which is an economic measure of consumer benefits: in practice the difference between what consumers are willing to pay for a product and the current market price.

Using a linear demand equation (based on a price elasticity of -0.7 and current market prices at Dutch supermarkets) the consumer surplus can be estimated as the area between the demand curve and the current market price in a plot of price versus quantity.

**3. Producer surplus**

The producer surplus represents the difference between the amount a producer of a product receives i.e. the market price and the minimum price at which the producer still would be willing to sell the product, in short the benefit for selling the product.

**References**

1. Kamphuis CB, de Bekker-Grob EW, van Lenthe FJ. Factors affecting food choices of older adults from high and low socioeconomic groups: a discrete choice experiment. The American journal of clinical nutrition. 2015;101(4):768-74. Epub 2015/04/04. doi: 10.3945/ajcn.114.096776. PubMed PMID: 25833974.

2. Lambooij MS, Veldwijk J, van Gils P, Mangen MJ, Over E, Suijkerbuijk A, et al. Consumers' preferences for freezing of meat to prevent toxoplasmosis- A stated preference approach. Meat science. 2018;149:1-8. Epub 2018/11/19. doi: 10.1016/j.meatsci.2018.11.001. PubMed PMID: 30448472.
