# Supplementary material for: A social cost-benefit analysis of two One Health interventions to prevent toxoplasmosis
Source: PLoS One. 2019 May 10;14(5):e0216615. doi: 10.1371/journal.pone.0216615 (PMC6510435; doi:10.1371/journal.pone.0216615)
Supplement: S5 Text — (DOCX) [file pone.0216615.s007.docx]

**S5 Text**

**Cat vaccination**

Using a disease dynamics model for *T. gondii* infections in cats and mice combined with an estimation of oocyst dose response, the impact of potential cat vaccination on human infections was assessed. Preliminary data revealed that it may not be feasible to carry out this intervention effectively, because reducing transmission to humans would require a cat vaccination coverage of more than 95% in populations of the order of 100 cats (and higher in larger populations), which is unlikely to be achieved and this was considering a hypothetical 100% effective vaccine. More details in Bonacic Marinovic et al. (submitted).
