# Supplementary figures and images for: A social cost-benefit analysis of two One Health interventions to prevent toxoplasmosis
Source: PLoS One. 2019 May 10;14(5):e0216615. doi: 10.1371/journal.pone.0216615 (PMC6510435; doi:10.1371/journal.pone.0216615)

S1 Fig

Assuming effectiveness 1%, lasting for 5 years in the minimum scenario

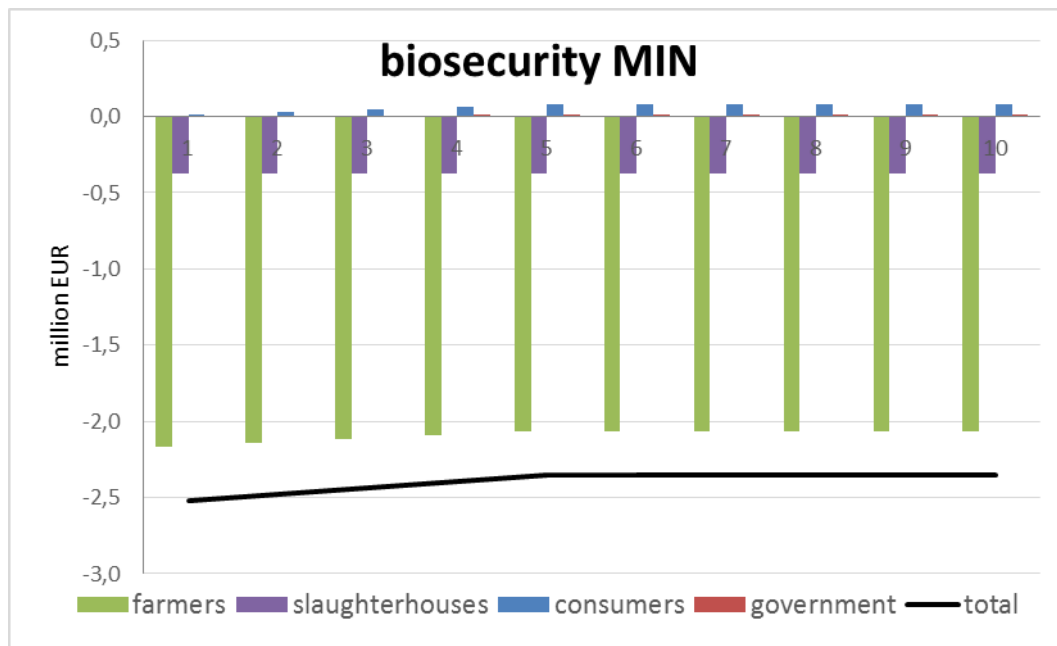

Supplement: S1 Fig — (PDF) [file pone.0216615.s008.pdf]

S2 Fig

Assuming effectiveness 1%, lasting for 5 years in the maximum scenario

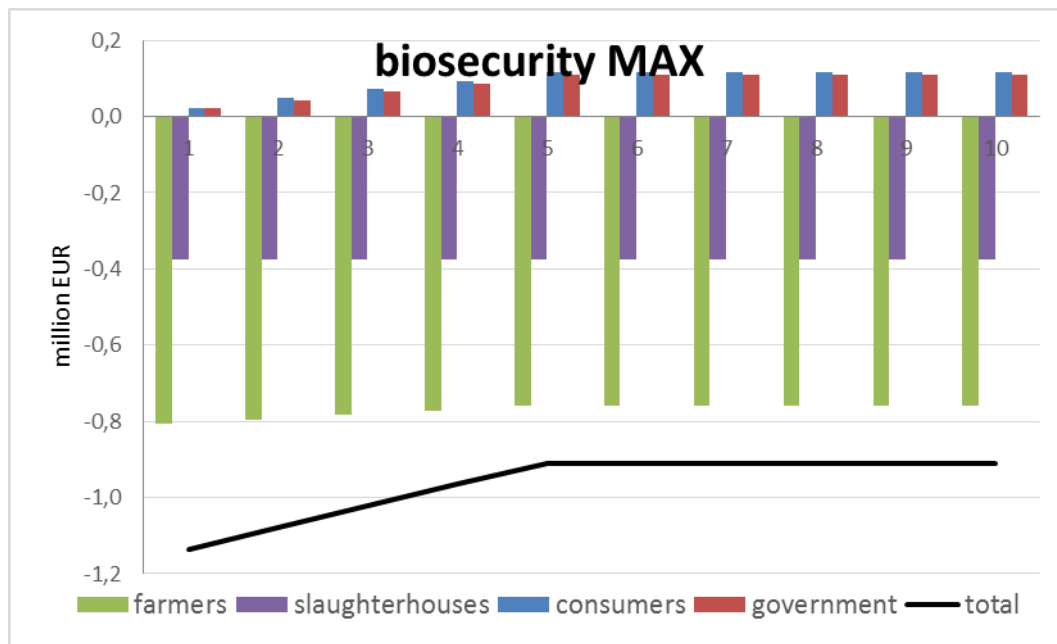

Supplement: S2 Fig — (PDF) [file pone.0216615.s009.pdf]

S3 Fig

Assuming effectiveness 1%, lasting for 10 years in the minimum scenario

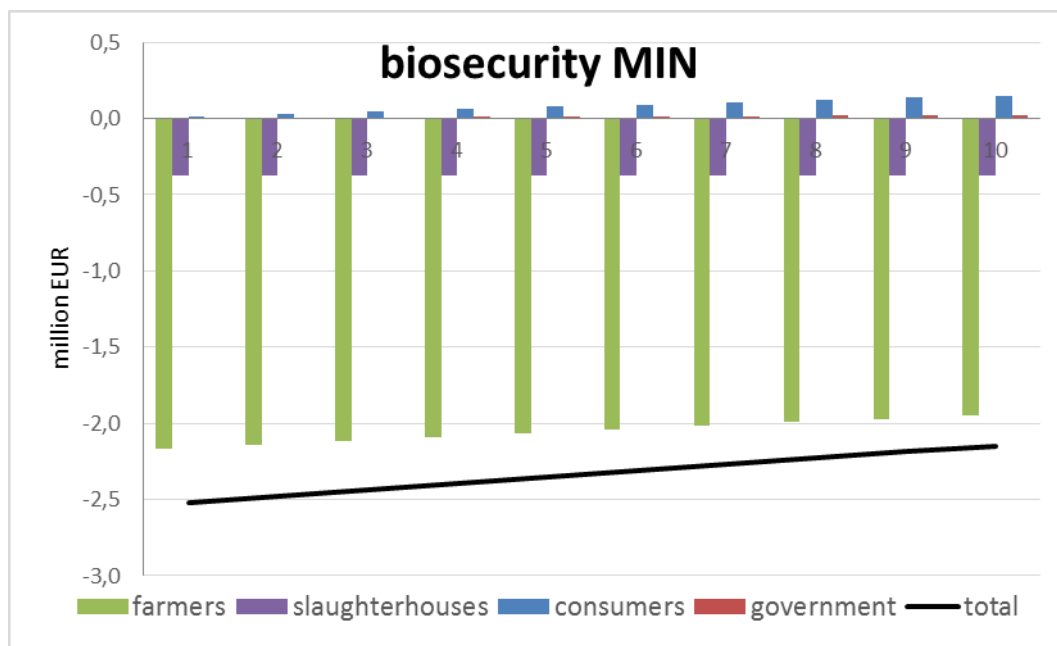

Supplement: S3 Fig — (PDF) [file pone.0216615.s010.pdf]

S4 Fig

Assuming effectiveness 1%, lasting for 10 years in the maximum scenario

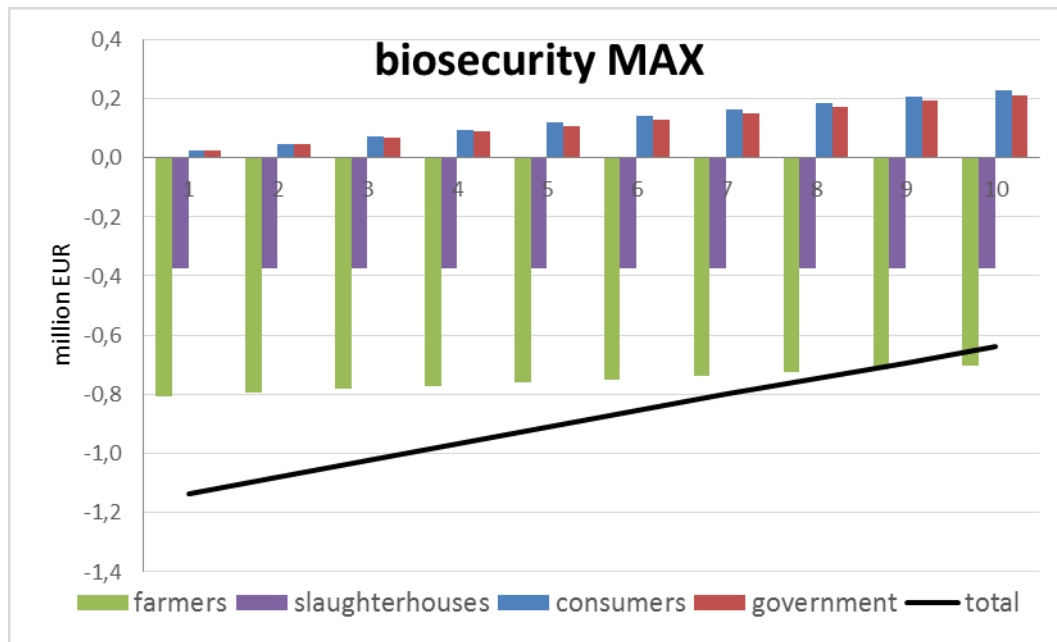

Supplement: S4 Fig — (PDF) [file pone.0216615.s011.pdf]

S5 Fig

Assuming effectiveness 10%, lasting for 10 years in the minimum scenario

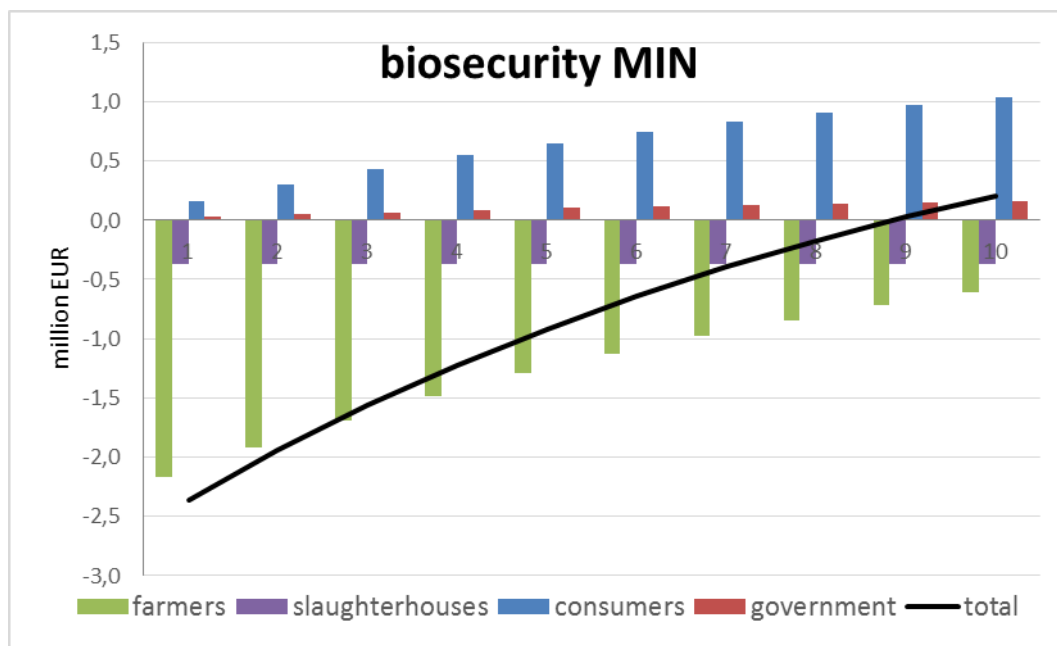

Supplement: S5 Fig — (PDF) [file pone.0216615.s012.pdf]

S6 Fig

Assuming effectiveness 10%, lasting for 10 years in the maximum scenario

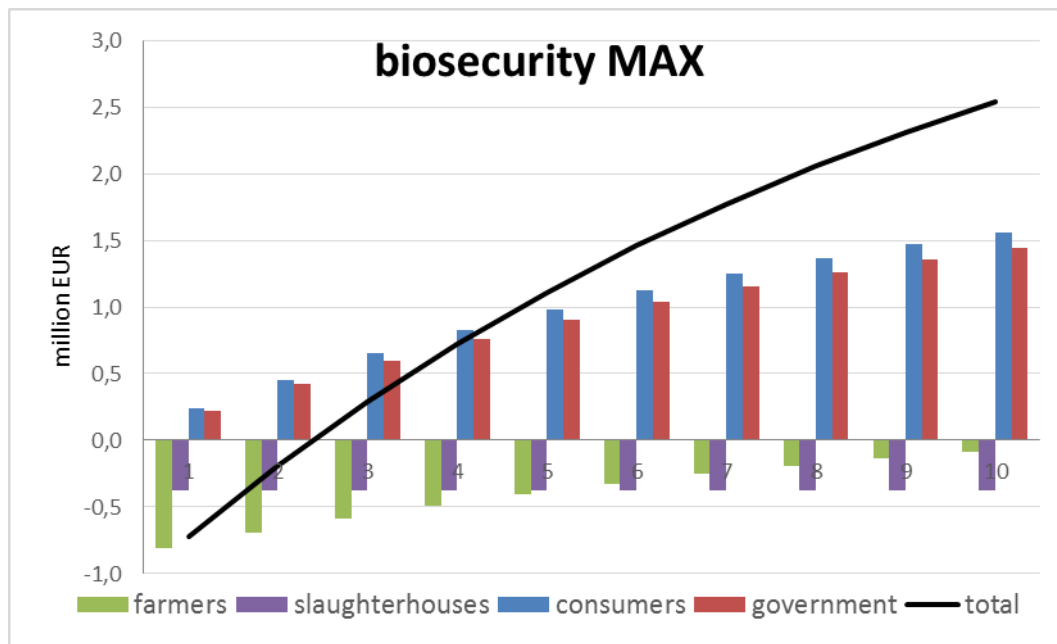

Supplement: S6 Fig — (PDF) [file pone.0216615.s013.pdf]
